# Chemotactic response with a constant delay-time mechanism in *Ciona* spermatozoa revealed by a high time resolution analysis of flagellar motility

**DOI:** 10.1242/bio.20137351

**Published:** 2015-01-08

**Authors:** Daisuke Miyashiro, Kogiku Shiba, Tahahiro Miyashita, Shoji A. Baba, Manabu Yoshida, Shinji Kamimura

**Affiliations:** 1 Department of Life Sciences, Graduate School of Arts and Sciences, The University of Tokyo, Tokyo 153-8902, Japan; 2 Shimoda Marine Research Center, University of Tsukuba, Shizuoka 415-0025, Japan; 3 Department of Biological Sciences, Faculty of Science and Engineering, Chuo University, Tokyo 112-8551, Japan; 4 Department of Advanced Biosciences, Graduate School of Humanities and Sciences, Ochanomizu University, Bunkyo, Tokyo 112-8610, Japan; 5 Misaki Marine Biological Station, Graduate School of Science, University of Tokyo, Miura, Kanagawa 238-0225, Japan

**Keywords:** Sperm chemotaxis, Flagellar motility, *Ciona intestinalis*, Constant delay model

## Abstract

During their chemotactic swimming toward eggs, sperm cells detect their species-specific chemoattractant and sense concentration gradients by unknown mechanisms. After sensing the attractant, sperm cells commonly demonstrate a series of responses involving different swimming patterns by changing flagellar beats, gradually approaching a swimming path toward the eggs, which is the source of chemoattractants. Shiba et al. observed a rapid increase in intracellular Ca^2+^ concentrations in *Ciona* spermatozoa after sensing chemoattractants; however, the biochemical processes occurring inside the sperm cells are unclear. In the present study, we focused on the timing and sensing mechanism of chemical signal detection in *Ciona*. One of the most crucial problems to be solved is defining the initial epoch of chemotactic responses. We adopted a high rate of video recording (600 Hz) for detailed analysis of sperm motion and a novel method for detecting subtle signs of beat forms and moving paths of sperm heads. From these analyses, we estimated a virtual sensing point of the attractant before initiation of motility responses and found that the time delay from sensing to motility responses was almost constant. To evaluate the efficiency of this constant delay model, we performed computer simulation of chemotactic behaviors of *Ciona* spermatozoa.

## INTRODUCTION

High efficiency in chemotactic responses and orientation toward eggs are the primary tasks of animal spermatozoa. For fertilization in many invertebrate animals, sperm cells need to detect their chemoattractants and sense their concentration gradients, which help sperm tails to steer for new paths of swimming to approach the eggs. In the case of chemotaxis of sea urchin spermatozoa, for example, the steering process has been investigated in detail (Cook et al., 1994; Ward et al., 1985; Eisenbach, 1999; Kaupp et al., 2006) and a theoretical approach to the swimming behavior using fluid dynamics has been also demonstrated (Alvarez et al., 2014). Because the patterns of swimming behavior during chemotaxis are similar among several animal species, it is assumed that there are a series of common mechanisms, from receiving external cue signals and transmitting intracellular signals to finally responding by controlling flagellar beat patterns. For example, in invertebrate animals, spermatozoa swim in constant spiral or circular orbits without the stimuli of attractants. When they sense chemoattractants, they demonstrate a series of responses to change flagellar beating patterns and swimming paths, i.e., short turning and straight swimming, and a recovery to the original swimming patterns drawing circular orbits, which gradually leads sperms toward eggs, the source of chemoattractants.

Regarding the detailed responses of spermatozoa, circular paths and orbit shifting during swimming and the symmetry–asymmetry switching of flagellar beats have been described thus far. Shiba et al. (Shiba et al., 2008) reported a fast increase in intracellular Ca^2+^ concentrations after sensing chemoattractant and a concomitant change in flagellar beat patterns with higher asymmetry. Thus, it was assumed that the concentration change of intracellular Ca^2+^ in *Ciona* spermatozoa was one of the most crucial events that trigger further downstream signal transduction inside sperm cells.

Our main question is related to the processes preceding the Ca^2+^ responses: how the extracellular attractants are perceived by chemoreceptors placed on the cell surface of spermatozoa, and how the mechanisms of signal transduction inside the cells trigger a change in flagellar beat pattern. Among the involved mechanisms, we focused on when and how spermatozoa sense the signals, which is one of the most crucial problems to understand the processes initiating chemotaxis.

From previous studies of chemotactic responses in *Ciona* spermatozoa, we assumed that the detected increase in Ca^2+^ concentration was just one event in a series of intracellular reactions after the sperm cells sense a concentration change of sperm-activating and -attracting factor (SAAF), a specific attractant for *Ciona* spermatozoa (Oishi et al., 2003; Oishi et al., 2004). According to the study by Shiba et al. (Shiba et al., 2008), the observed timing of Ca^2+^ responses corresponded to a point where sperm cells swam through a point in which SAAF concentration in their swimming orbits was minimum, which is also the most distant point from the attractant source. However, there have been no clear explanations on how the Ca^2+^ response was triggered at this specific point. Assuming that there may be a sub-threshold level of Ca^2+^ concentration that was not detected, we assumed that it was also possible that the actual intracellular signal transduction responses in spermatozoa started before the spermatozoa passed through the minimum concentration point.

One of the simplest explanations for chemotactic responses was recently proposed to describe the reaction of sea urchin spermatozoa to speract, an oligo amino acid attractant derived from eggs (Guerrero et al., 2010). After a careful analysis of cGMP-dependent Ca^2+^ signaling inside sperm cells, it was suggested that spermatozoa are equipped with a type of delayed timer that transmit the cue attractant signals perceived by sperm cell surface receptors to the intracellular downstream mechanism of chemical reactions. Such a programmed timer with approximately 0.1–0.2 s of delay is hypothesized to trigger the downstream Ca^2+^ responses just on a suitable timing of chemotactic responses, as observed in sea urchin sperm (Guerrero et al., 2010; Böhmer et al., 2005). Based on the delayed timer model, it has been shown that the chemotaxis of sea-urchin spermatozoa swimming both in two-dimensional circular and three-dimensional helical paths can be explained (Friedrich and Jülicher, 2007).

In the present study, we used a similar delayed timer model to determine whether or not we can detect changes in sperm motion preceding the Ca^2+^ responses. We assumed that there is a threshold of responses in the fluorescent dye Ca^2+^ indicator and in an image acquiring system used to detect Ca^2+^ burst signals. Therefore, we expected a minute change in spermatozoa motions or shapes at any beat phase of flagellar motility preceding the Ca^2+^ signals. In addition, we expect a change in spermatozoa swimming paths or head positions if any mechanical response or flagellar shape changes in a sub-threshold level are occurring inside sperm cells, as long as the sperm motility was observed under low Reynolds number conditions. Thus, we used a high-rate video recording setting (600 Hz) and time resolution (1.7 ms) to perform a detailed analysis of sperm motion which in the case of *Ciona* spermatozoa can occur at a beat frequency of <60 Hz, corresponding to a beat cycle of >17 ms. In addition, we performed novel fine analysis of beat forms and swimming paths of spermatozoa to detect subtle signs occurring during the sub-threshold level of Ca^2+^ responses to SAAF. From such experiments, we expect to determine an exact point of SAAF sensing that should precede the initiation of motility responses.

## RESULTS AND DISCUSSION

### Definition of sperm position relative to the attractant source

**Fig. 1.**
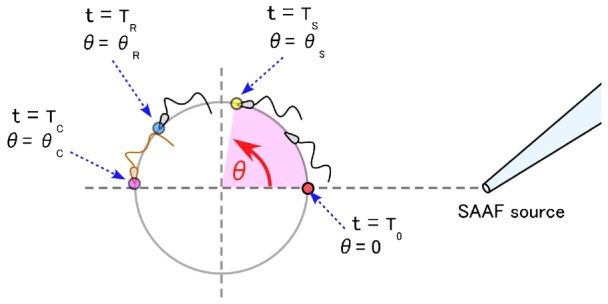
Diagram illustrating the definition of angle (θ) and time (T). T_0_ and θ = 0: when the head of swimming spermatozoa passes the line connecting center of the swimming orbit and the SAAF source (right side). T_s_ and θ_S_: time and the angle when the spermatozoa sense the concentration change of SAAF, and a series of reaction of chemotactic signal transduction is initiated. T_R_ and θ_R_: when the first mechanical response of flagellar beat appears. T_C_ and θ_C_: when Ca^2+^ response is observed. The Ca^2+^ responses were not observed in the present study, but we assumed that the rise of Ca^2+^ concentration occurs at the same timing as reported previously.

On observation of a chamber under the microscope, *Ciona* spermatozoa was seen to swim in circular paths with almost constant radii and swimming velocities. To express the head positions of swimming spermatozoa, we used angle (θ) instead of usual XY-coordinates as illustrated in Fig. 1. First, we defined the nearest position of the swimming orbits from the source of attractant, SAAF; hereafter, we refer to this specific position and time as θ = θ_0_ (θ_0_ = 0), and t = T_0_, respectively. After the spermatozoa swim through the point of θ = θ_0_, we assumed that the cells would experience three types of events. First, they would sense changes in SAAF concentration at point θ = θ_S_, and time t = T_S_. During this step, we were not interested in unraveling the exact mechanisms used by sperm cells to find cues of concentration changes. Second, after T_S_, the first mechanical response triggered by sensing SAAF should appear in the flagellar waveforms or the head position of spermatozoa at point, θ = θ_R_, and time, t = T_R_. The period between T_S_ and T_R_, where θ_S_<θ<θ_R_ was assumed to be a latent period when the intrinsic chemotactic responses of spermatozoa have already been initiated, but we cannot observe them as apparent changes of flagellar wave shapes. Finally, we defined the point where Ca^2+^ signals can be detected, at the point, θ = θ_C_, and time, t = T_C_, which, in *Ciona*, corresponds to the most distant point from the attractant source (Shiba et al., 2008).

**Fig. 2.**
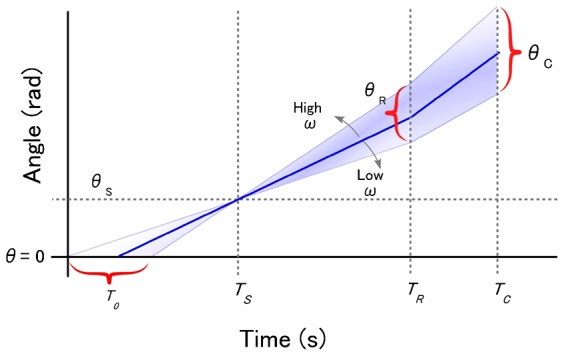
Diagram illustrating our working hypothesis. Definition of θ and T are as shown in Fig. 1.

To interpret a linear relationship between angular velocity and angle (position) that will be given in our results, we first introduce a working hypothesis as follows. Because we could observe that the rate of sperm swimming is constant before it started any mechanical responses, where T_0_<t<T_R_, and θ_0_<θ< θ_R_, there would be a linear relationship between θ and t, and therefore, we would be able to obtain a regression line representing the relation between t and θ from experimental data (Fig. 2). From this analysis, the angular velocity (ω, rad/s) can be directly calculated from the slope of the line obtained for each sperm cell. We should also observe statistical variations caused by individual differences in swimming velocities, path radius, and angular velocities (ω) per cell. Thus, from the observed experimental variations, we should expect to determine the exact point of θ_S_ and θ_R_ based on our working hypothesis of constant delay model as follows: i) sensing point, θ_S_, is fixed relative to the SAAF source; ii) latent period, T_R_–T_S_, is constant in every sperm cell; iii) latent period for Ca^2+^ responses, T_C_–T_R_, is constant in every sperm cell. This hypothesis also suggests that there will be some statistical variations of other parameters such as θ_R_, θ_C_, and T_0_ (Fig. 2), which would be derived from the variations in swimming speed of individual sperm cells.

To determine T_R_–T_S_ and T_C_, we used the statistical variations in spermatozoa swimming speeds. At time T_R_, the response point at θ = θ_R_, spermatozoa with the faster swimming velocity (ω) will reach the farther point in a constant delay time of latency, T_R_–T_S_; thus, the relation can be expresses by the equation:
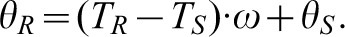
(1)

By calculating the slope and y-intercept of the regression line of correlation of ω versus θ_R_, we can determine T_R_–T_S_ and θ_S_, respectively. Similarly, we can determine θ_S_ and T_C_–T_S_, which we also expected to be constants, as shown in Fig. 2, using the following equation:
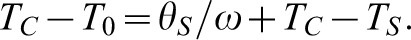
(2)

By calculating the slope and y-intercept of the regression line of the correlation of 1/ω versus T_C_–T_0_, we can determine T_C_–T_S_ and θ_S_, respectively. As described below, the obtained correlations are given in Fig. 5. During the statistical analysis of swimming speed variations, the accuracy to find the exact values of θ_R_ and T_R_ was crucial. We used two independent methods to determine θ_R_ and T_R_: the analysis of trajectory shift of sperm head motion and the detection of flagellar shape changes.

### Determination of the response time, T_R_, from the analysis of sperm head trajectories

At first, we analyzed the trajectories of sperm head motions in a resting state without SAAF stimuli, where *Ciona* spermatozoa repeated relatively regular and circular paths of swimming. The images recorded at 600 fps clearly show that the trajectories of head position were in circular orbits superimposed with small elliptic motions with amplitudes of approximately 2 µm; the motions were synchronized with the flagellar beats (30–60 Hz), as shown in supplementary material Fig. S1. We fitted the head motions to the following empirical equation:
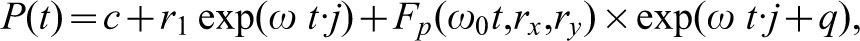
(3)where, *P(t)* is the position of sperm head expressed in complex coordinates, *j* is an imaginary unit, ω is the velocity of circular orbit, *ω_0_* corresponds to the velocity of head swinging (flagellar beat frequency), *F_p_* represents a transcribed form of periodic functions of 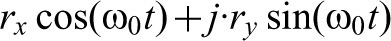, and *q* is a constant. *r_x_* and *r_y_* are the minor and major axes of the head's elliptic motions, respectively.

The final residuals from the observed trajectories after fitting the equation (3) showed a Gaussian distribution as shown in supplementary material Fig. S1F. This indicated that the observed trajectories of sperm head motions could be described by the equation (3) without any systematic biases. The final residual noise (standard deviation) we observed here was 0.50 µm. The noise was not derived from the Brownian motion of sperm heads, but from the accuracy with which we calculated the brightness centroids of sperm head images using an image analyzing software (Bohboh Soft, Tokyo, Japan). These accuracies were almost equivalent to the pixel size we used (0.73 µm/pixel). It should be noted that the head position of *Ciona* spermatozoa could be decided with pixel size precision in the present study. This precision was reasonable because assuming that the size of a sperm head and the length of flagellar tail are approximately 3 µm and 50 µm respectively, then the estimated mean distance of Brownian movement during one frame time period of 1/600 s can be estimated to be 0.05 µm or less (D>0.1 µm^2^/s). More importantly, it indicated that we could detect any subtle changes in the head positions of spermatozoa; thus, the flagellar beat changes under flow condition of low Reynolds numbers, with a high time resolution (1/600 s).

**Fig. 3.**
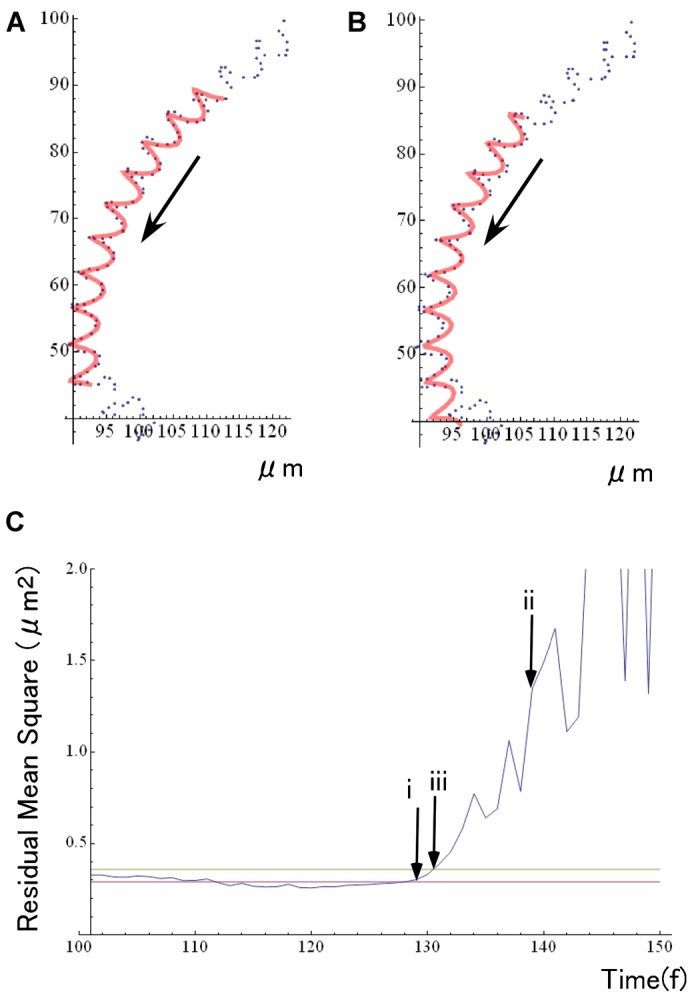
Determination of T_R_ from the analysis of the head trajectory. (A,B) Observed center positions (center of brightness) of the heads of swimming spermatozoa (blue dots) and an empirical fitted curve (red lines) using the equation (3). (C) Plots of the summation of residual mean squares, i.e., squares of distance between the estimated and observed head position. At the time of arrow-i, which corresponds to the data shown in (A), we could fit the data of head position to an empirical equation with residuals less than a threshold of deviation (yellow line, Material & Methods). At the time of arrow-ii, which corresponds to the data shown in (B), it is clearly shown the residuals have already increased over the threshold. We determined the point arrow-iii in (C), the exact point for the residuals to cross over the threshold, the point where the sperm head position began sudden deviations from the predicted trajectory curves, which we assume to be T_R_, corresponding to the time when the first response after chemotactic stimuli started. Red line: mean residuals (summation of mean squares) obtained during the 120 frames prior to the video frame used for the analysis.

Using the method of equation fitting, we determined the point when the first sign of sperm motility change was detected, i.e., θ_R_ and T_R_. We then compared the observed head positions with those predicted from the trajectory by the empirical equations as follows. First, we calculated the residuals between real head positions and empirical equations (Fig. 3A,B), subsequently we checked the point at which the sum of squares of these residuals increased over a critical value defined by the error of measurements (cf. Materials and Methods, Fig. 3C). We assumed this detected point corresponded to the time when the head positions started to be deviated from a predictable resting state, which gave the time T_R_, when initial detectable changes in flagellar beats occurred.

### Determination of the response time, T_R_, from a novel wave form analysis

We performed out an additional analysis to determine the deviating points from the steady resting state of swimming as follows. First, we obtained the frame by frame position of each flagellar segment recorded from microscope images of *Ciona* spermatozoa using a software design for the analysis of flagellar waveforms (Bohboh Soft, Tokyo, Japan). Then, we converted all the segment positions into a new coordinate plane by putting the flagellar base at the coordinate origin and by placing the flagellar tip on the x-axis. Supplementary material Fig. S2 represents such examples. As long as the spermatozoa repeat stable waveforms, all the flagellar waveforms will fall inside an enveloping area represented by the superimposed blue lines in supplementary material Fig. S2. If there is a slight change in waveforms, we expected to detect it as a new line deviating from the enveloped area (red lines in supplementary material Fig. S2). We assumed that the time when the red waveforms are formed corresponds to T_R_.

To perform a quantitative evaluation of the beating waveforms, we introduced a new parameter named wave-shape distance defined by the following equation:
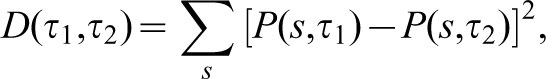
(4)where *τ_1_* and *τ_2_* are the time of image recording, *s* represents the position of each segment along flagella, i.e., distance from the flagellar base, and *P* is the position of each segment in the two-dimensional coordinate as shown in supplementary material Fig. S2. *D(τ_1_,τ_2_)* is a quantity representing the difference of two wave forms between the time *τ_1_* and *τ_2_*.

**Fig. 4.**
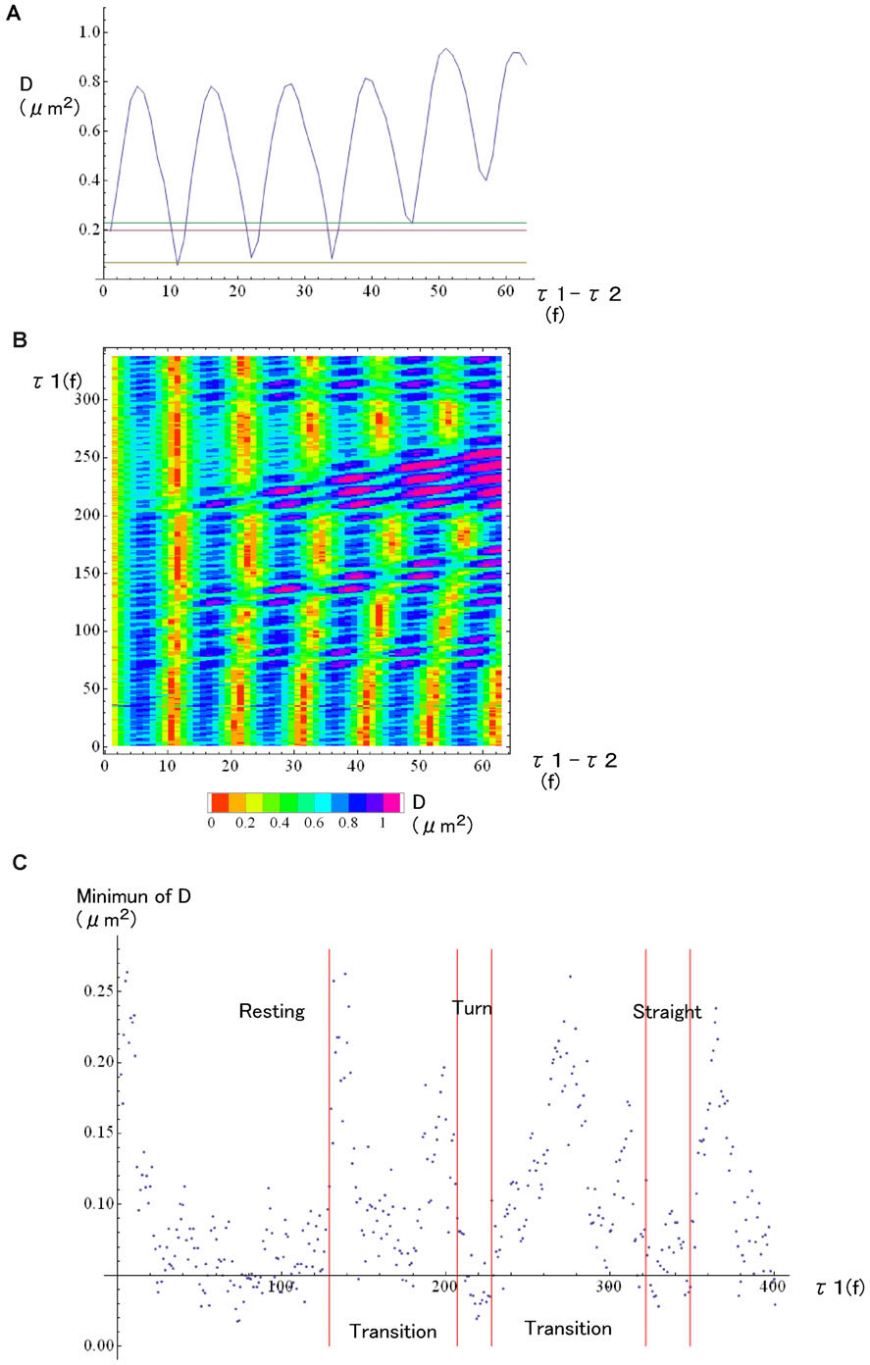
Detection of T_R_ from the waveform analysis. (A) Time course of calculated *D(τ_1_,τ_2_)*, the waveform distance at τ_2_ from a fixed point at *τ_1_*. Red and yellow lines indicate the threshold *D* values reflecting waveform deviations without attractant stimuli, i.e., normal fluctuations of waveforms, obtained by the analysis with time resolution of 1/600 s and 1/1200 s, respectively. Green line indicates the value of threshold *D* that we used to detect the change of beating pattern. (B) Pseudo-color image indicating the value of *D(τ_1_,τ_2_)* in a plane *τ_1_* versus *τ_1_–τ_2_*. From the data shown in (B), we extracted the value of minimum D and are plot against *τ_1_* as in (C).

If flagella beat in a steady state regime repeating regular waveforms, then any waveform represented in supplementary material Fig. S2 can be completely superimposed with another waveform in the same phase of beat. In such case, the wave-shape distance *D(τ_1_,τ_2_)* should be zero. We should also expect a synchronized variation of the values of *D(τ_1_,τ_2_)* with the beat phase of sperm flagella, i.e., when *τ_1_* = *τ_1_*+2nπ/ω_0_ or when *τ_2_* = *τ_2_*+2nπ/ω_0_, where n is an integer and ω_0_ is the phase velocity of flagellar beat (Fig. 4A). It is also to be expected that when comparing the waveforms between two different times with the same beat phases, the value of *D(τ_1_,τ_2_)* should be zero when *τ_1_* = *τ_2_*+2nπ/ω_0_. More precisely, we can define the waveform synchrony with an accuracy of beat phases of 0.31–0.42 rad from the time course variations of *D(τ_1_,τ_2_)* because we recorded the motion of *Ciona* spermatozoa at a 30–60 Hz beat frequency (beat period, 17–33 ms) with 600 fps (time resolution, 1.67 ms).

Fig. 4 shows examples of the performed waveform analysis. As shown in Fig. 4A, the wave-shape distance *D(τ_1_,τ_2_)* varied depending on *τ_1_*–*τ_2_*, the time difference between two waves. Fig. 4B indicates another example of cyclic variation of *D(τ_1_,τ_2_)* depending on both *τ_1_* and *τ_1_*–*τ_2_*. From such plots, we could compare two waveforms of spermatozoa with high phase accuracy and we could detect T_R_ with a time resolution of 1.67 ms. Instead of using the waveform coordinates shown here, we also tried similar methods to compare waveform phases using different parameters, i.e., a shear-angle curve that reflects the angle of flagellar shaft, the magnitude of microtubule sliding in flagellar axonemes, and the flagella curvature correlated with the magnitude of axonemal bending (data not shown). However, we have chosen the present waveform analysis because it uses direct data from recorded images in a more accurate manner and had lower analytical noises than the other two mentioned methods.

Under hydrodynamic condition of low Reynolds number (approximately 10^−6^ for swimming *Ciona* spermatozoa), any inertia during swimming motion is negligible and the active forces generated by the moving spermatozoa are instantaneously balanced with external viscous drag forces. It also indicates that the observed head motions should be occurring simultaneously with the flagellar shape changes. The values of T_R_–T_0_ portrayed in supplementary material Fig. S3 show that the time delay of the spermatozoa mechanical response from the position θ_0_ on the swimming orbits are quite consistent in two independent analyses; the analysis of head motion trajectories (Fig. 3; supplementary material Fig. S1) and of wave-shape distance (Fig. 4; supplementary material Fig. S2). Due to the good agreements between these two methods, we used the results of wave-form analysis for the further detailed investigations because of its smaller variations and higher time resolutions.

The bend angle of flagella was not symmetric, but in many cases there were larger bends on one side comparing to the other. The bends on the larger side were usually outside the circular swimming paths and are called principal-bends (P-bends) (Gibbons and Gibbons, 1972). The bends on the other side are called reverse-bends (R-bends). From the detailed analysis of motion, we found that the timing to the initial point of mechanical responses T_R_, always corresponded to the time when the bends are maximal on either the P- or R-bend sides. These new features we found here could reflect an unknown switching mechanism inside motile flagella, where new beating patterns are always initiated as a new bend formed at the proximal ends of the flagella. In other words, spermatozoa cannot freely change the waveforms at any time; this change depends on their beat phases. It would imply that the mechanism of beat form regulation of spermatozoa is not fully optimized for instantaneous responses to the concentration changes of chemoattractant. How the localization of attractant receptors on sperm cells is related to this restriction of bend regulation would be a next important question to be addressed.

It also led us to another conclusion that we could not avoid the fluctuations within ±1/2-beat period (π rad of phase) in T_R_ values that we obtained in the present study. Details of the switching mechanisms of beat pattern are not clear yet, but for the accuracy of our analytical method as well as for the spermatozoa showing the chemotactic swimming, the problems of timing errors of approximately 10 ms in T_R_ (corresponding to the half beat period of flagella) cannot be avoided due to the constraints in the mechanism of waveform switching. This error may become one of the main factors of fuzziness in the chemotactic behaviors in swimming spermatozoa, but their biological aspects are unclear.

### Sperm response under SAAF stimuli

The observed changes in swimming path under SAAF stimuli were almost the same as those reported previously (Shiba et al., 2008). There were two short states showing different curvatures of swimming orbits, a state of turning, and that of straight-swimming. These states appeared after a stable state of regular circular path of swimming (resting state) that was usually observed without a SAAF gradient. Using the waveform analysis described above, we could define the starting and ending points of each state of steady swimming with high time resolution (Fig. 4C). We also found there were unstable transients before spermatozoa came into new states as described below.

During transients from stable swimming to another state, there were irregular waveforms with high *D(τ_1_,τ_2_)* values compared with other steady states. These transients corresponded to the period when we could observe two mixed bends in one flagellum; a new bend propagating from the proximal base to distal tip along the flagellar shaft with an old bend being diminished at the tip end. The period of such unsteady transients varied from 50–80 ms to several beat periods (<4.5 beat cycles). The observed minimum periods of transients appeared to be the time required for old waves to be replaced by new ones, the time for wave propagation. Periods of observed transients are summarized in Table 1. In the following analysis, to determine the time of SAAF sensing (T_S_) and mechanical response (T_R_), we carefully analyzed the recorded microscope images during chemotactic swimming of spermatozoa.

**Table 1.**
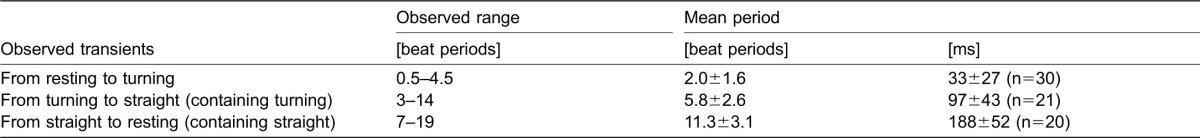
Periods of transition between different wave form patterns

In typical cases under the condition of SAAF stimuli, spermatozoa showed a cyclic change in waveforms, such as resting → turning → straight swimming → resting → and so on. However, as shown in Table 1, the variation of each transient period was not small. In some cases, the resting steady state was so short that we could not fit it to any empirical functions for head trajectories. In other cases, a new cycle of reactions started during transitions or even in the middle of a recovery period. Thus, to determine T_R_ in the following analysis, we deleted such complicated cases and chose data which had stable resting states for at least four beat periods of flagella.

In the present study on *Ciona* spermatozoa, we were able to divide swimming behaviors into discrete steady states of different waveform parameters. Completely different interpretations, however, were suggested in the case of chemotactic behavior in sea urchin spermatozoa, where a model including continuous and smooth conversions of beating asymmetry of sperm flagellum was used in the theoretical approaches by Friedrich and Jülicher (Friedrich and Jülicher, 2007). The difference of swimming behavior suggested that *Ciona* sperm may have the pathways of signal transduction circuit that make the sharp recognition of chemoattractant and the steep Ca^2+^-dependent responses of beating asymmetry. Further investigations are required to understand the difference of sperm responses in these two chemotactic systems.

Based on the definition of time and position of sperm heads shown in Fig. 1 and the working hypothesis shown in Fig. 2, we first obtained T_R_, the time when the first apparent change of waveforms was found during chemotactic swimming. Then, we calculated the head positions θ_R_, with distribution in a range of 0.5–3 rad (1.89±0.76 rad), as shown in supplementary material Fig. S4A. According to the studies by Shiba et al. (Shiba et al., 2008), the position of Ca^2+^ burst (θ_C_) was approximately θ_C_∼π (3.14 rad) in our definition (Fig. 1). There could be two possible explanations for the difference of θ_R_ and θ_C_. One is that Ca^2+^ bursts in an undetected sub-threshold magnitude were already occurring before the changes in the beat form. The other possibility is that Ca^2+^ bursts could be the phenomenon occurring downstream the beat-form changes as shown in our working hypothesis (Fig. 1). As suggested by the dynamic model of sperm chemotaxis by Böhmer et al. (Böhmer et al., 2005) and Guerrero et al. (Guerrero et al., 2010), the wave forms of sperm flagella seems to be determined not directly by the concentration of Ca^2+^ at a given time, but by a preceding history of concentration changes would be crucial. In addition, Shiba et al. (Shiba et al., 2008) also showed that the beating asymmetry appeared not to be not directly corresponding to the detected Ca^2+^ concentrations, in particular, during the recovery states after chemotactic responses. Further investigations would be required to provide correct answers to the question, how the concentration change of intra-sperm Ca^2+^ is correlated to the flagellar waveforms during the chemotactic responses.

**Fig. 5.**
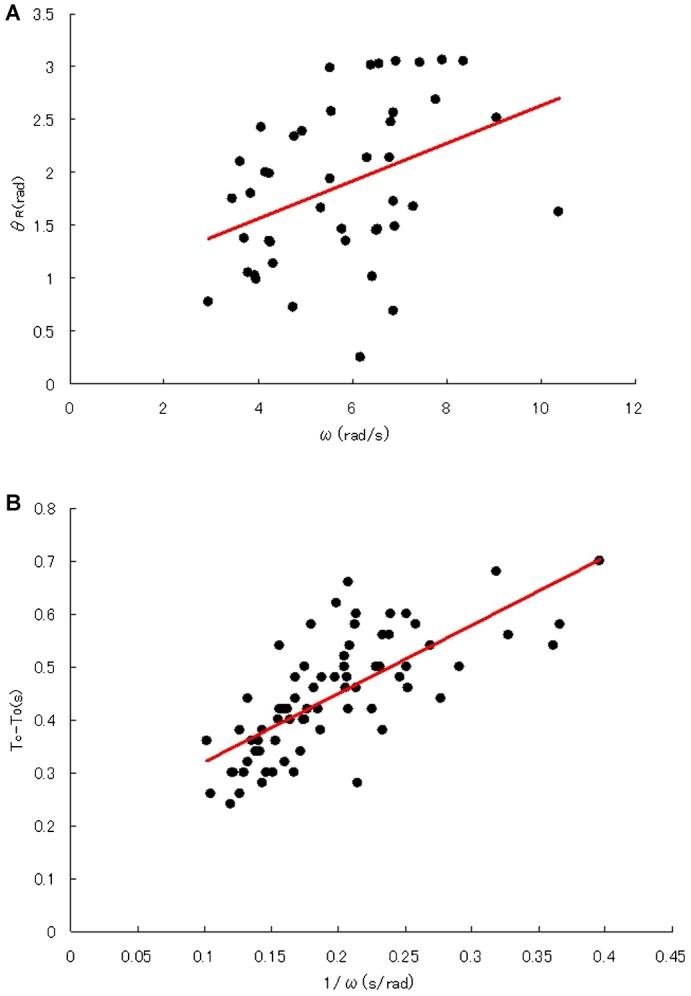
Analysis of θ_R_ and θ_S_ based on our working hypothesis of chemotactic responses. (A) Diagram showing the correlation between θ_R_ and ω, the angular velocity of sperm swimming. According to the equation (1) in the text, delay time (T_R_–T_S_ = 0.18 s), and the sensing point (θ_s_ = 0.85) are obtained from the line slope and y-intercept, respectively (n = 44, correlation coefficient = 0.388). (B) Diagram showing the correlation between T_C_–T_0_ and the inverse of swimming velocity (1/ω). According to the equation (2) in the text, the sensing point (θ_s_ = 1.3 rad) and the delay time (T_C_–T_S_ = 0.19 s) are obtained from the line slope and y-intercept, respectively (n = 72, correlation coefficient = 0.727).

Next, we focused our analysis on the difference between θ_S_ and θ_R_, the delay of response after a SAAF sensing point, which could not be directly observed (Fig. 1). As shown in supplementary material Fig. S4A and Fig. 5A, there was some dispersion of θ_R_ that could have come from the fluctuation in the beat forms of swimming spermatozoa. According to the equation (1) and our working hypothesis (Fig. 2), we obtained 0.18 s and 0.85 rad for T_R_–T_S_ and θ_S_ from the slope and y-intercept of regression line, respectively (Fig. 5A). According to the equation (2), we also obtained values of 1.3 rad and 0.19 s for θ_S_ and T_C_–T_S_ from the slope and y-intercept of the regression line in Fig. 5B, respectively. Because of unknown reasons there was a larger scattering of data in ω versus θ_R_ in Fig. 5A than those in T_C_–T_0_ versus 1/ω in Fig. 5B, we assumed that θ_S_ obtained using the equation (Eqn 2) would reflect more accurate value of real the SAAF sensing position of spermatozoa. Thus, we concluded that some signals coming from the change of SAAF concentration in the medium triggered a mechanism to induce Ca^2+^ burst and changed the beating patterns of sperm flagella with a delay of approximately 0.18–0.19 s, which approximately corresponds to a position (θ_S_) of approximately 1.3 rad (Fig. 1).

**Fig. 6.**
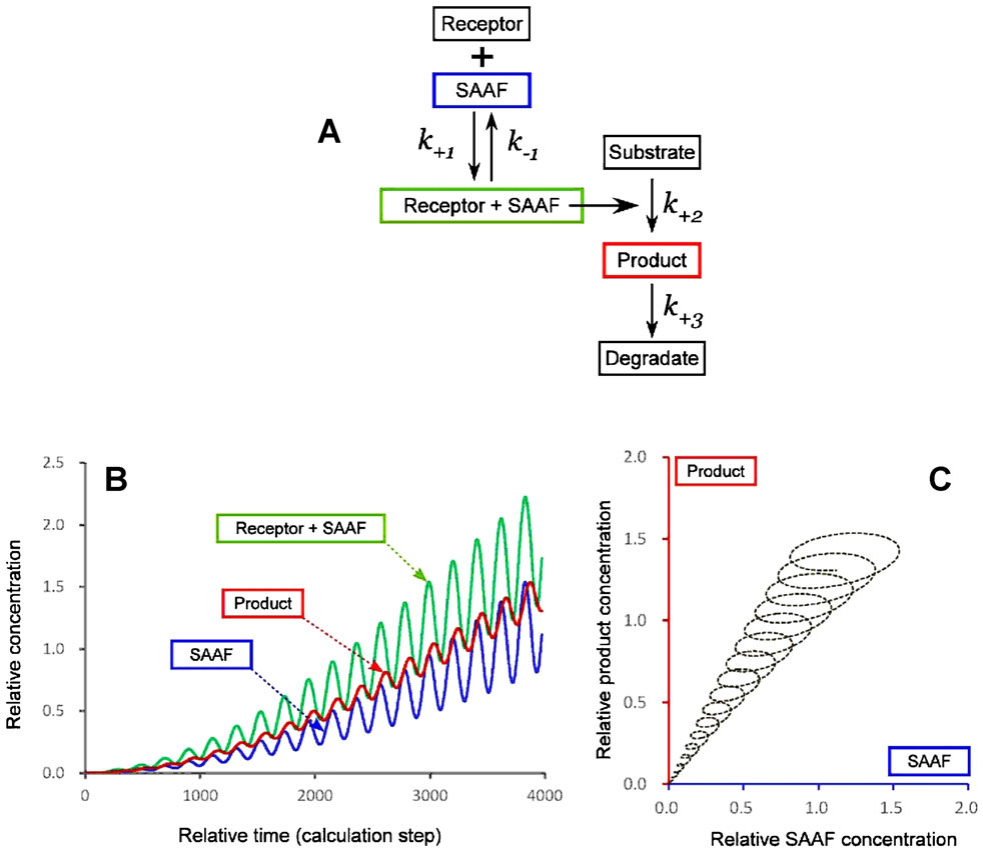
Kinetics model of signal transduction to explain the constant delay in chemotactic response. (A) A minimum set of kinetics model, where a receptor activated by the chemoattractant (SAAF) accelerates downstream chemical reaction (k_+2_) that catalyzes the production of a second messenger (Product). (B) Simulated time course of product formation when the sinusoidal modulation of SAAF concentration was given. For the tentative calculations, k_+1_/k_−1_, k_+2_/k_+3_, substrate concentration, and receptor concentration of 0.25, 10, 1, and 16 were used, respectively. Although this model is composed of a tentative minimum set of kinetics parameters, we can mimic well the chemotactic responses with a constant delay phase of π/2 (1.6 rad). (C) Diagram showing the correlation between the concentrations of SAAF and the product. The time delay can be kept almost constant in a relatively wide range of SAAF concentrations.

From the analysis stated above, we obtained the values, θ_s_ = 1.3 rad, T_R_–T_s_ = 0.18 s, and T_c_–T_s_ = 0.19 s. The point of θ_s_ = 1.3 rad on the sperm swimming path corresponds to the place where the sperm experiences the most negative gradient of SAAF concentration, i.e., the maximum decreasing rate of SAAF concentration. Our analysis suggests a mechanism of switching in which a high rate of concentration decrease triggers a Ca^2+^ spike and induces the motions of *Ciona* spermatozoa into smaller circular orbits with a constant time delay of approximately 0.18 s. As for the mechanisms of signal transduction to detect the SAAF concentration, further biochemical investigations are required. For example, a simple kinetics model that can explain the phenomenon is shown in Fig. 6. In this minimum model, the most critical point is to sense the changing speed of SAAF concentration even at high concentrations. Such a feature may be accomplished by fine balancing between the timing of concentration changes and the biochemical rates of signal transductions occurring inside *Ciona* spermatozoa. As for supporting evidence, it has been shown that the beating pattern of sea-urchin spermatozoa was not directly regulated by the concentration Ca^2+^ alone, but rather by its rate of change (Alvarez et al., 2012). The kinetics model shown here would suggest a similar mechanism is included in the beat form regulation. To clarify other details, it will be required to perform careful experiments revealing the dynamic responses of each step in the unknown chemotactic reactions of spermatozoa (Fig. 6B).

In the case of chemotaxis of sea urchin spermatozoa (Kashikar et al., 2012), it was assumed that there is an integration time of 100–200 ms required for the chemotactic network to response, which makes smooth averaging of chemoattractant concentrations, but at the same time it limits the time-resolution of gradient sampling by swimming spermatozoa. This would be also the case in *Ciona*, where spermatozoa are sensing the gradients and fluctuations of chemoattractant that are exposed to swimming spermatozoa exactly synchronized with the head yawing during flagellar beating as shown in Fig. 3A and supplementary material Fig. S1. In our analysis, it was shown that the point (T_S_) for swimming spermatozoa to detect the attractant gradient was corresponding to the phase of motion in an area with the most negative gradient (Figs 1, 6). Even if spermatozoa are sensing the averaged concentrations of chemoattractant of the last 100 ms, an expected time delay would be included in T_R_–T_S_, or T_C_–T_S_ in our analysis. Thus, our data would not be completely inconsistent with the previous model of sensing averaged attractant concentrations.

Another remaining open question is to describe in detail the SAAF concentration gradient sensing mechanism. If spermatozoa are equipped with a type of differential sensor that is placed on sperm head and other tail regions, we can expect that spermatozoa may easily respond to the gradient of chemicals. Thus, we investigated how the observed T_S_ (time to sense SAAF) were correlated with the sperm orientations, the flagellar waveforms, or the directions of sperm tails. However, as far as our collected data were concerned, we could not find any fixed correlation. We concluded that spermatozoa can sense SAAF concentration regardless of the beat forms of spermatozoa, flagellar waveforms and cell orientations. Therefore, the model to detect concentration gradient at two different points on sperm cells would be unlikely. Alternatively, as suggested by double pulse experiments (Kashikar et al., 2012), a model of the temporal sensing of attractant would be more likely. In the case of sea-urchin chemotaxis, signals accumulation by receptors placed along flagella (Cardullo et al., 1994) would be working. In the case of *Ciona* we used, the localization and properties of actual SAAF receptors were not clarified yet.

### Simulation of chemotactic response based on the constant delay model

From detailed analysis of spermatozoa swimming under the condition of attractant stimuli, we determined the SAAF sensing point (θ_S_), and the delay time for response (T_R_) to be approximately 1.3 rad, and 0.18 s, respectively. Following the SAAF sensing, Ca^2+^ burst, and a series of different states of beat forms, turning, straight-swimming occurred before recovering to the original resting state of normal beating. It ultimately induced shifting of the circular orbits of sperm swimming and resulted in the access toward eggs. To discuss the efficiency of this constant delay model in a quantitative way, we carried out model calculations by Mathematica (ver. 8, Wolfram Research).

**Fig. 7.**
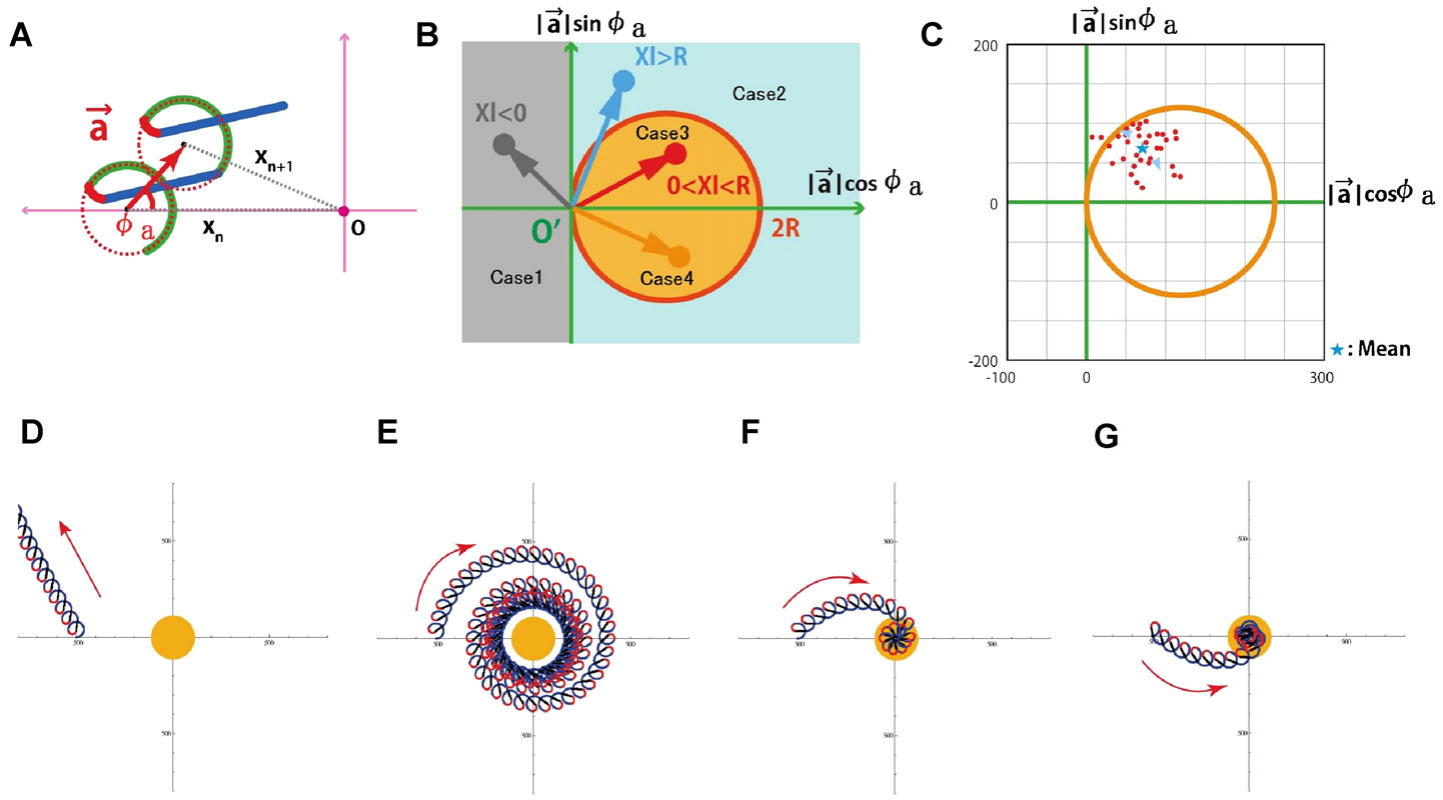
Calculation of chemotactic efficiency. (A) Definition of chemotactic vector (*a*, red arrows) as a connecting line from the *n*-th center of the circular orbit of sperm swimming (X_n_) during resting state to the *n+1*-center of the next orbit (X_n+1_) appearing after a series of chemotactic responses, i.e., resting → turning → straight-swimming → recovery to resting states. The center origin of coordinates features the egg (attractant source). *Φ*_n_ is the angle between *a* and [0, X_n_]. (B) Diagram showing the vectors *|a|·cosΦ* versus *|a|·sinΦ* to categorize the chemotactic efficiencies. (C) The same diagram described with a real µm scale. (D–G) Simulated swimming paths of spermatozoa in Cases 1–4, respectively. The central yellow circle is an egg with a 120-µm diameter. If the tip end of vector *a* is placed on a circle described as 2R = *|a|/cosΦ_a_* (orange circle), the spermatozoa always keep swimming with a constant distance from the egg. Vectors place outside and inside the circle correspond to diverging and converging swimming orbits, respectively. Here we show four examples of the chemotactic vector (Cases 1–4) that represent typical patterns of sperm swimming as shown in D–G, respectively. As shown in C, actual *Ciona* spermatozoa showed the vectors included in the category Case 3 with some fluctuations.

For the calculations, we simplified the swimming path of chemotactic responses to be composed of three steady states (resting, turning and straight) without any states of transients as shown in supplementary material Fig. S5. In order to evaluate how the actual fluctuations in the response delay time affects the behavior, we included random variations in T_R_–T_S_ of comparable size to our observations (supplementary material Fig. S5A). To describe the results of chemotactic responses, we defined the change in swimming orbits by a vector of chemotaxis, *a*, that describes the net shift of swimming orbits using two independent parameters, |*a*| and *Φ*_*a*_ (Fig. 7A). This vector, *a*, is varied depending on θ_S_, T_R_–T_S_, and also on the time periods of steady and transition states. By the summation of vectors determined under various conditions as shown in Fig. 7F, we could simulate the global behavior of swimming sperm. supplementary material Fig. S5B represents the case in fluctuation-free condition for comparison.

The most efficient chemotaxis would correspond to the case where the chemotactic vectors are exactly pointing toward the egg center. From our calculations, we could classify four cases depending on the efficiency as shown Fig. 7B. Case 1 describes the situation where the vectors are pointing to the gray zone in Fig. 7B of *cosΦ_a_*<0, which was determined by a certain combination of the delay time of response, T_R_–T_s_, and the angular velocity of sperm swimming, ω. It occurs typically in the case when T_R_–T_S_>0.35 s or swimming angular velocity is too high. Because the swimming path of spermatozoa is pointing in wrong directions, spermatozoa cannot approach eggs and will fail in chemotaxis (Fig. 7D). Thus, the delay time and swimming velocity should be regulated in a suitable range for a successful chemotaxis. Case 2 is another extreme example of failure, where T_R_–T_S_ and ω are larger than some critical values resulting in |*a*|/*cosΦ_a_*>2R. In this case, spermatozoa can approach the egg, but are finally trapped in a circular orbit at a fixed distance from the egg. For successful chemotaxis, it is necessary that the vectors point to the zone of Case 3 or 4 in Fig. 7B.

Using the observed θ_R_, the position where *Ciona* sperm flagella responded to SAAF (supplementary material Fig. S4A) and ω, the swimming angular velocity (Fig. 5A), we performed simulations to mimic actual cases. The observed chemotactic vectors and the estimated path of chemotactic swimming are shown in Fig. 7C–G and supplementary material Fig. S5, respectively. Fig. 7A indicates the presence of some statistical fluctuations in *Ciona* spermatozoa; however, almost all the chemotactic vectors in actual *Ciona* spermatozoa were pointing to the zone of Case 3, showing stable and efficient chemotaxis. Even if there are some fluctuations in the response of an individual spermatozoon, i.e., T_R_–T_S_ varies in some range, the chemotaxis with Case 3 vectors shows stable responses that were not essentially different from the case without fluctuation (supplementary material Fig. S5). We repeated 100 similar simulations but *in silico* spermatozoa showed efficient chemotaxis finally arriving to the egg in 20 s. Such helical swimming paths of chemotactic behavior is quite similar to those described in the study of Friedrich and Jülicher (Friedrich and Jülicher, 2007).

In the Case 3 or 4 in Fig. 7B, the most efficient and ideal behavior of sperm chemotaxis that show the fastest approach to the eggs, should be with the chemotactic vectors of *cosΦ_a_* = 1, i.e., *Φ_a_* = 0. However, the actual observed angles were scattered around the area in Case 3 with *Φ_a_* approximately π/4 with unknown reasons. The situations where *Φ_a_* = 0 would correspond to the case of chemotactic behaviors with no time delays in the responses, or those with high acceleration of signal transduction (*k_+2_* in Fig. 6A). We speculate that the actual cases where *Φ_a_* = π/4 may affect the robustness of behaviors in environmental perturbations, e.g., decrease the swimming speed by lowering the temperature, high viscous medium, and fluid flows. Further detailed simulations and comparisons incorporating other factors should be performed to find the correct answers to the question of why the observed *Φ_a_* was π/4. In addition, simulations in three-dimensional space where spermatozoa may show different orbits and speeds of swimming, as shown by Corkidi et al. (Corkidi et al., 2008), would be required.

### Conclusion

In the present study, using the recording system of *Ciona* sperm motility with 600 fps and precise methods to detect fine changes in flagellar beat forms, we were able to define a point (T_R_) of the first response to occur prior to the intracellular Ca^2+^ bursts. Further analysis of the statistical variations in response time based on our working hypothesis, suggest that there is a mechanism to sense the change in SAAF concentration (T_S_) 0.18 s before T_R_. We conclude that by proposing the mechanism revealed here, i.e., that spermatozoa senses the π/2 delay in the temporal change of attractant concentration, and then modify the flagellar beat patterns after a constant delay time; this would be one of the essential mechanisms to regulate the sperm chemotaxis. The delay time model has been already suggested by Böhmer et al. (Böhmer et al., 2005) and Guerrero et al. (Guerrero et al., 2010); however, the actual sensing point and delay time are determined for the first time based on the detailed observations of swimming patterns with high time resolutions. From our simulation, it was also shown that the simplified model of sperm responses with a constant time delay after signal sensing could explain the efficiency of sperm chemotaxis.

## MATERIALS AND METHODS

### Materials

*C. intestinalis* was collected in Aburatsubo Bay (Kanagawa, Japan) and maintained in an aquarium under a constant light condition until use to prevent spontaneous spawning. *Ciona* sperm specimen was obtained from sperm ducts by dissection and was stored on ice before use. Sperm motility was observed in artificial seawater (ASW) containing 462 mM NaCl, 9 mM KCl, 10 mM CaCl_2_, 48 mM MgCl_2_, and 10 mM HEPES–NaOH (pH 8.2). We used synthesized SAAF, described previously (Oishi et al., 2003; Oishi et al., 2004).

### Analysis of sperm swimming paths and flagellar waveforms

Collected *Ciona* semen was diluted 10^4^–10^5^ times in ASW containing 1 mM theophylline (Sigma), which activated sperm motility by increasing intracellular cAMP (Yoshida et al., 1994). An aliquot of suspension containing activated sperm was placed into a one-side open chamber for observation made of strips of silicone rubber (0.5-mm thick) and cover slips, the surface of which were previously coated with 1% BSA to avoid nonspecific sperm adhesion. To develop an attractant gradient in the chamber, a 1% agar solution containing 1 µM SAAF was enclosed at the tip of a glass micropipette (tip diameter, 50–100 µm). From 0.5 to 3 min after inserting the micropipette at an open side of the observation chamber, images of the sperm around the micropipette tip were recorded. Sperm images were observed under a phase-contrast microscope (Olympus BX51) with a 20× objective (Olympus UPlan FINH) equipped with the power LED stroboscopic illumination system. Movie recording of swimming spermatozoa was performed under the flashing illumination of 100-µs duration (Shiba et al., 2008) synchronized with a high-speed CCD camera (HAS-220; Ditect, Tokyo, Japan). Using the sperm motion analyzing software (BohbohSoft, Tokyo, Japan), the XY-coordinate points corresponding to the center of sperm head as well as flagellar segments along each flagellar tail (0.5 µm unit length) were determined frame by frame in recorded swimming spermatozoa. These coordinate data were used for further details calculations of wave forms.

### Fitting empirical curves to sperm head trajectories

From the obtained high-speed video images, the luminosity centroid of sperm heads were determined with the image-processing function equipped in the Bohboh, which automatically calculated the head centers by fitting two-dimensional Gaussian distributions. As we used a phase objective for bright contrast which had little contrast reversing, the obtained image center of sperm heads should have been corresponding to the center of head mass (center of summed refractive index).

As shown in Fig. 3 and supplementary material Fig. S1, the observed trajectories of sperm heads were composed of a circular path and small zigzag lines. We first fitted the trajectory to a circle (supplementary material Fig. S1A) and obtained the subtracted data. Then, we fit the subtracted data to an elliptical orbit with rotating axis (supplementary material Fig. S1C). After the fitting (supplementary material Fig. S1E), the remaining residuals showed Gaussian distribution (supplementary material Fig. S1F). Although the least-squares method was fundamentally used for the fitting after manually choosing initial parameters using the function FindMinimun of Mathematica, it was difficult to execute automatic calculations to find the global minimums. Such calculation was carried out on the basis of the sperm head locus data for approximately 20 cycles, and the empirical equation (3) of the swimming trajectory was obtained. Although the remainder of Gaussian distribution had a slight distortion, it did not influence the finding of the final head position, i.e., in the case shown in supplementary material Fig. S1, e.g., remaining errors were 0.00556±1.44 µm and 0.0436±1.08 µm in x and y directions, respectively.

### Wave-distance analysis

If beating flagella repeat exactly the same waveforms, the wave-distance parameter (D) as defined by the equation (4) would be minimum in the same beat phases. Since the wave-distance parameter should depend on the time resolution of our experiment, it can be described by the following equation:
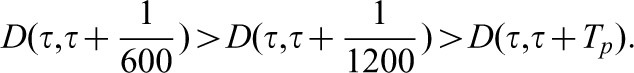


Here, τ is time when we observed waveforms, and T_p_ is the exact beat period of flagella that could be defined by infinite temporal resolution. Using the equation (4), we can calculate the wave-distance for any pairs of waveforms between τ1 and τ2. Then, for any beat periods and phases, if any subtle changes of waveforms were there, we could detect them as sudden changes of D(τ_1_,τ_2_) with the temporal resolution of 1/600 s. Using another set of waveforms of a sea urchin spermatozoa recorded with a super-high-speed camera (6,000 fps, data not shown), we tested how the detectability of D(τ_1_,τ_2_) depended on the beat phases of flagella. It was clarified that the detectability to find sudden change of waveforms as shown in Fig. 4A,B, as well as in supplementary material Fig. S1, did not depend on the beat phases, e.g. P-bend, R-bend, or any other intermediate phases. Therefore, it is suggested that this new parameter, wave-distance, used here can be applied to any other cases of flagellar or ciliary motility in order to detect subtle shifts of waveforms.

## Supplementary Material

Supplementary Material
