# Supplementary material for: Chemotactic response with a constant delay-time mechanism in Ciona spermatozoa revealed by a high time resolution analysis of flagellar motility
Source: Biol Open. 2015 Jan 8;4(2):109–18. doi: 10.1242/bio.20137351 (PMC4365479; doi:10.1242/bio.20137351)
Supplement: Supplementary Material [file supp_4_2_109__index.html]

Chemotactic response with a constant delay-time mechanism in Ciona spermatozoa revealed by a high time resolution analysis of flagellar motility — Chemotactic response with a constant delay-time mechanism in Ciona spermatozoa revealed by a high time resolution analysis of flagellar motility — Supplementary Material 

# Chemotactic response with a constant delay-time mechanism in *Ciona* spermatozoa revealed by a high time resolution analysis of flagellar motility

## bio.20137351 Supplementary Material

**Files in this Data Supplement:**

- Supplementary Material - Daisuke Miyashiro et al. doi: 10.1242/bio.20137351
