# Supplementary material for: Chemotactic response with a constant delay-time mechanism in Ciona spermatozoa revealed by a high time resolution analysis of flagellar motility
Source: Biol Open. 2015 Jan 8;4(2):109–18. doi: 10.1242/bio.20137351 (PMC4365479; doi:10.1242/bio.20137351)
Supplement: Supplementary Material [file supp_bio.20137351_bio.20137351-s1.pdf]

Biology Open (2015) 000, 1–10 doi:10.1242/bio.20137351

Supplementary Material
Daisuke Miyashiro et al. doi: 10.1242/bio.20137351

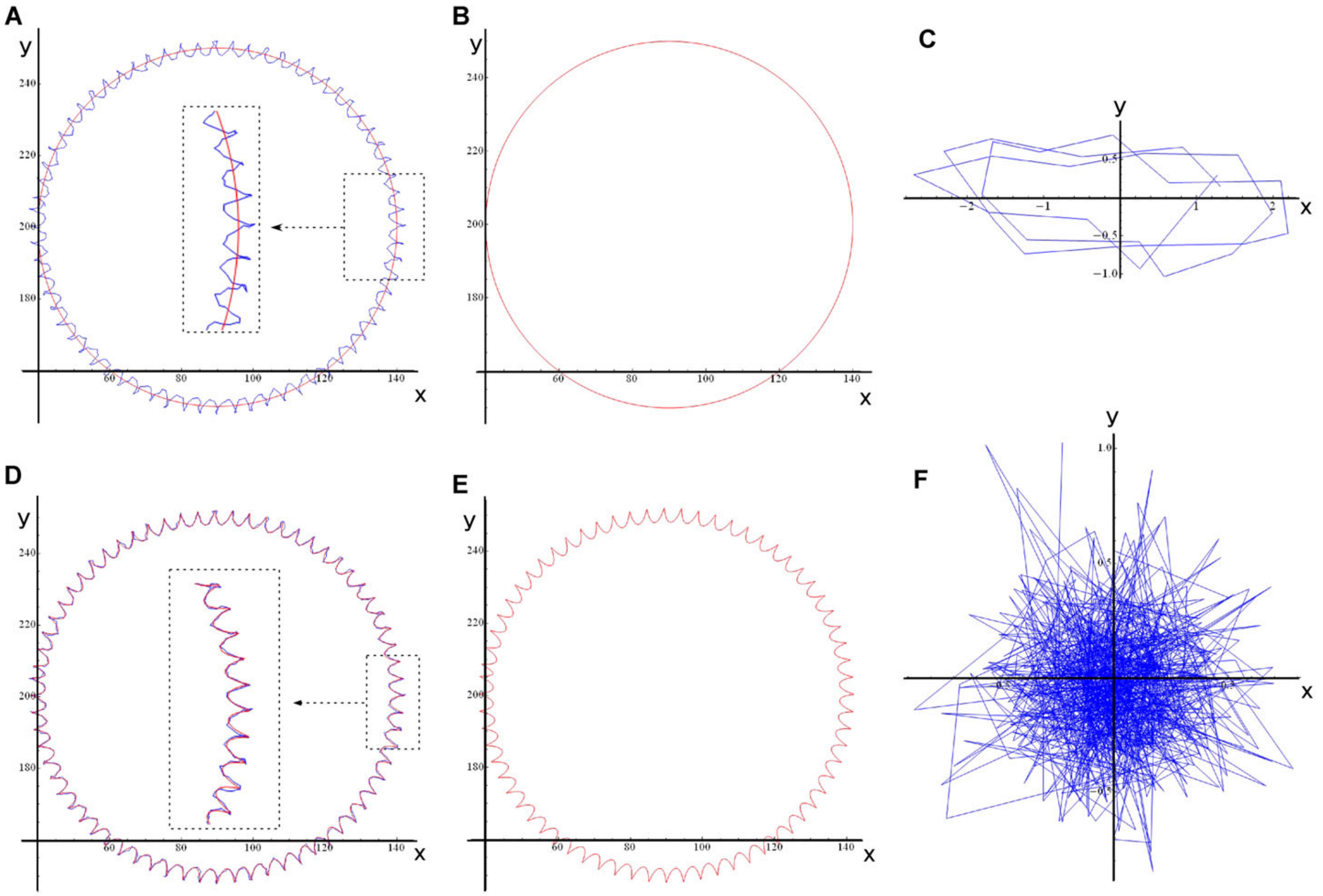

Fig. S1. Fitting the trajectory of sperm head motions to empirical equations. (A,B) Example of the first fitting of observed trajectory data of a sperm head (blue curves) to a circular orbit (red line). (C) Residuals after fitting, i.e., the difference between the observed and estimated curves, are shown during three continuous cycles of the motion of head swinging, from which the secondary fitting curve of elliptic traces of head motion was empirically obtained. (D,E) Secondary fitting of observed trajectory data of a sperm head (blue curves) to a circular orbit plus head swings with an empirical elliptic traces (red). (F) Final residuals after the second fitting showing random Gaussian distribution without any systematic biases. The unit of axes is μm.

Biology Open (2015) 000, 1–10 doi:10.1242/bio.20137351

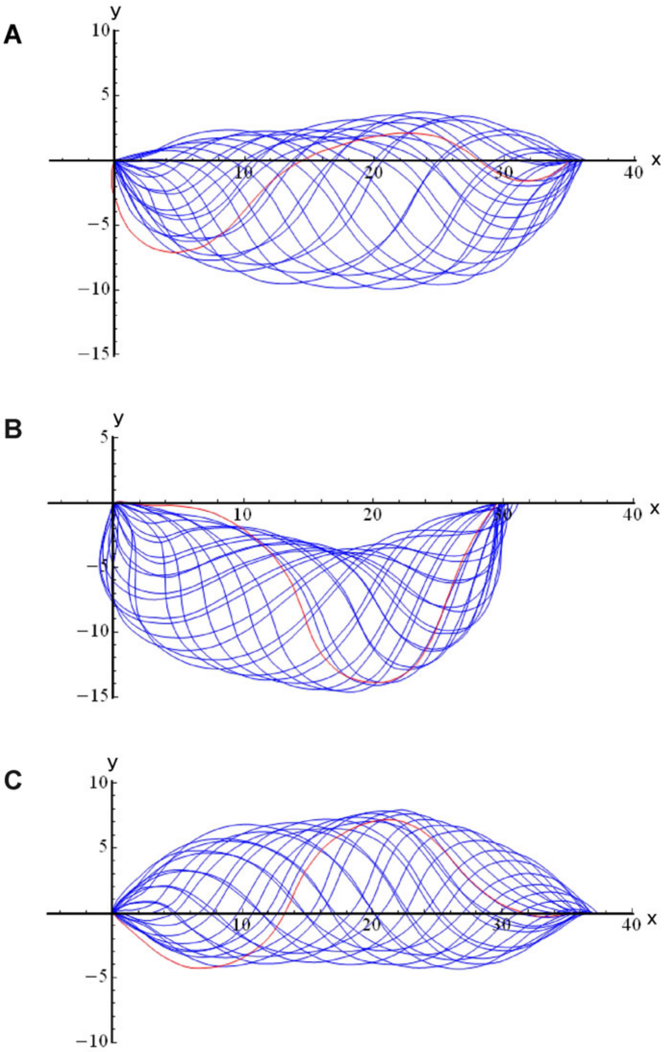

Fig. S2. Example showing the waveform analysis. We described the waveforms by placing each flagellar segment on a new coordinate plane, where the proximal base of sperm flagella is placed on the coordinate origin and the tip end of flagella placed on the x-axis. Blue lines indicate waveforms during two beat periods when we could observe steady-state beating before T_R. Red lines represent waveforms deviations from steady-states, corresponding to the point of time, T_R. A, B, and C are the waveforms during resting state without attractant stimuli, turning state, and the state showing straight-swimming, respectively. The unit of axes is μm.

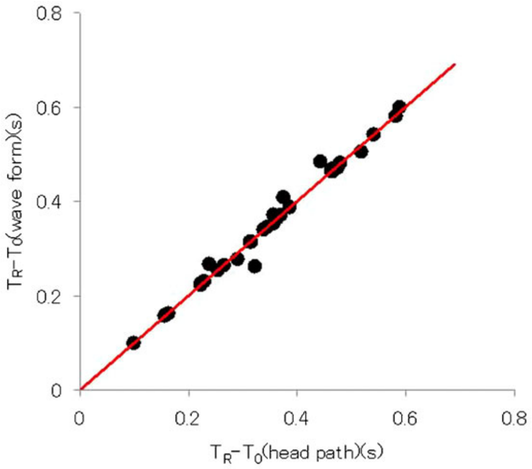

Fig. S3. Comparison of T_R–T_0 obtained by two different methods. All the values of T_R both in the trajectory analysis of sperm heads (x-axis, Fig. 3; supplementary material Fig. S1) and in the wave-shape analysis (y-axis, Fig. 4; supplementary material Fig. S2) were shown. Consistent results were obtained in both analyses. (n=33, correlation coefficient=0.992).

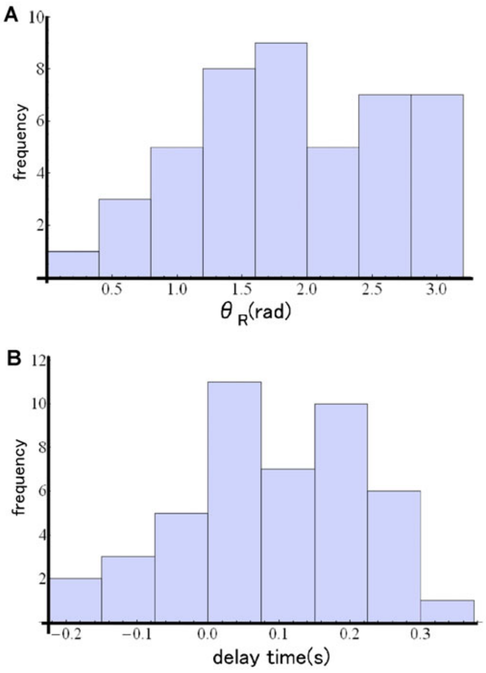

Fig. S4. Histograms showing the distribution of obtained response point (θ_R) and delay time (T_R–T_S). (A) Histogram of θ_R, 1.89 ± 0.76 rad. (B) Histogram of estimated T_R–T_S, 0.093±0.127 s.

Biology Open (2015) 000, 1–10 doi:10.1242/bio.20137351

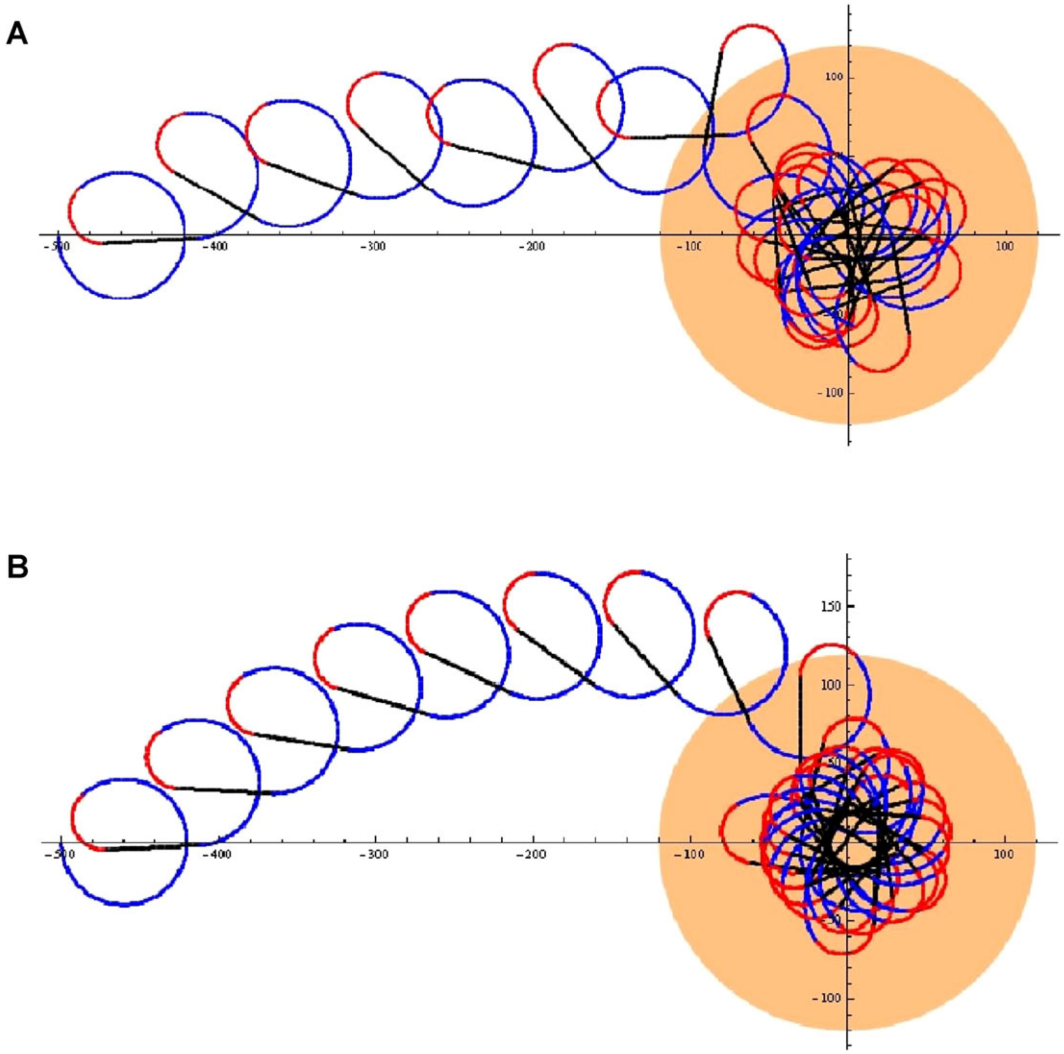

Fig. S5. Swimming path simulation of *Ciona* spermatozoa during chemotaxis. Swimming paths drawn with blue, red and black lines are corresponding to the sperm motion in states of resting, turning, and straight swimming, respectively. The orange circle represents an egg, the attractant source. (A) Chemotaxis simulate with $\theta_S = \pi/2$ (sensing point) and $T_R – T_S$, of 0.093 s$\pm$0.127 s. (B) Chemotaxis simulate with $\theta_S = \pi/2$ (sensing point) and $T_R – T_S$ of 0.093 s (delay time) without statistical deviations. The unit of axes is μm.

Biology Open
